# Heme Mediates Cytotoxicity from Artemisinin and Serves as a General Anti-Proliferation Target

**DOI:** 10.1371/journal.pone.0007472

**Published:** 2009-10-28

**Authors:** Shiming Zhang, Glenn S. Gerhard

**Affiliations:** Weis Center for Research, Geisinger Clinic, Danville, Pennsylvania, United States of America; Mental Health Research Institute of Victoria, Australia

## Abstract

Heme (Fe2+ protoporphyrin IX) is an essential molecule that has been implicated the potent antimalarial action of artemisinin and its derivatives, although the source and nature of the heme remain controversial. Artemisinins also exhibit selective cytotoxicity against cancer cells *in vitro* and *in vivo*. We demonstrate that intracellular heme is the physiologically relevant mediator of the cytotoxic effects of artemisinins. Increasing intracellular heme synthesis through the addition of aminolevulinic acid, protoporphyrin IX, or transferrin-bound iron increased the cytotoxicity of dihydroartemisinin, while decreasing heme synthesis through the addition of succinyl acetone decreased its cytotoxic activity. A simple and robust high throughput assay was developed to screen chemical compounds that were capable of interacting with heme. A natural products library was screened which identified the compound coralyne, in addition to artemisinin, as a heme interacting compound with heme synthesis dependent cytotoxic activity. These results indicate that cellular heme may serve a general target for the development of both anti-parasitic and anti-cancer therapeutics.

## Introduction

Heme (Fe^2+^ protporphyrin IX) is a ubiquitous and essential tetrapyrrole molecule that performs a wide array of fundamental physiological functions, including oxygen transport, electron transfer, and transcriptional regulation. Heme is highly redox active and is potentially toxic in its free form, which is present at sub-micromolar concentrations in most cells [1]. Heme has been implicated in the anti-malarial mechanism of artemisinin (ART) and its derivatives which are toxic to malaria parasites at nanomolar concentrations [2]. We have recently shown that redox active heme is the most efficient and kinetically favorable activator of artemisinin in vitro relative to oxidized ferric heme (hemin), inorganic ferrous iron, ferric iron, hemoglobin, or methemoglobin [3] which show only limited reactivity under certain experimental conditions [3], [4], [5], [6]. Artemisinin molecules contain an endoperoxide linkage that is required for anti-parasitic activity. The endoperoxide bond accepts an electron presumably from heme, which results in drug activation, subsequent bond breakage, and the formation of an alkoxyl radical in the artemisinin molecule [7]. Heme-artemisinin adducts have been identified in malaria-infected mice [8], a strong indication that the C4 radical of artemisinin is relevant to the antimalarial activity of artemisinin.

In addition to acting as an anti-malarial agent, artemisinins are also selectively cytotoxic to cancer cells in vitro, including drug- and radiation-resistant cancer cell lines [9]. Artemisinins have also been shown to be effective against cancer in rodent models [10], [11], [12] and have begun to be used in human cancer patients [13], [14], [15]. Despite the significant efforts to delineate their anti-malarial mechanism, how artemisinins exert their cytotoxic effects has only recently begun to be investigated. Artemisinins have been reported to inhibit tumor lymphangiogenesis by suppression of vascular endothelial growth factor C in a mouse lung cancer cell line [16], block estrogen receptor expression in a human breast cancer cell line [17], and increase calcium levels and activate p38 in a human lung cancer cells [18].

We hypothesized that the initial interaction with heme served as a common mechanism of activation for artemisinins in exerting cytotoxic effects against both malaria parasites and cancer cells. Cancer cells have an increased capacity to synthesize heme [19], as well increased requirements for iron from increased synthetic demand due to higher rates of proliferation [20]. Aminolevulinic acid (ALA), a heme precursor derived from the condensation of glycine and succinyl-CoA in the mitochondria by the enzyme ALA synthase which is the rate-limiting step in the heme synthesis pathway and is feedback inhibited by heme, has been used as a pharmacological agent to increase heme synthesis to generate photosensitive heme precursors such as PPIX for photodynamic therapy in treating cancers [21], [22].

We demonstrate that pharmacological inhibition of heme synthesis decreases the cytotoxicity of artemisinins, while stimulation increases cytotoxicity. We also developed an in vitro high-throughput heme interaction assay to screen a library of natural products that identified several compound(s) with reactivity towards heme that exhibited exquisite molecular specificity. One compound, coralyne, was highly cytotoxic with dependence upon heme synthesis. The heme interaction high-throughput screening assay may serve as a useful tool to identify new compounds against cancer, malaria, and other parasites. Heme may be an especially attractive target molecule for the development of a new molecular class of chemotherapeutic agents.

## Methods

### Chemicals

Dihydroartemisinin (DHA) and sodium artesunate (ATS) were a generous gift from Dafra Pharma N.V. (Belgium). All other chemicals were obtained from Sigma-Aldrich (St. Louis, MO). Hemin, PPIX, ART, DHA, and ATS and peroxide compounds were prepared in DMSO (used immediately or stored at −20°C for later use). Succinyl acetone (SA), ALA and holotransferrin (HTF) were prepared in 0.1 M phosphate buffer, pH 7.0. Sodium dithionite solution was freshly prepared for each assay in 80 mM phosphate buffer, pH 7.0 containing 20% DMSO.

### Heme Interaction Assay

The following reagents were added in sequence into standard clear flat bottom polystyrene 96 well microplate (Costar) wells: hemin (0.125 mM in DMSO, final 20 uM), chemical compounds (in DMSO at 40 uM), dithionite (200 mM in 80 mM phosphate buffer pH 7.0, containing 20% DMSO, final 160 mM). The molar ratio of compound∶heme was 2∶1. Parallel wells for each compound were prepared that did not contain hemin. The plate was shaken 10 times in the microplate reader before reading at 415 nm using a Spectra MAX250 Microplate Reader (Sunnyvale, CA, USA). The absorbance of wells without heme was subtracted from the corresponding wells with hemin for each microplate. The positive control contained DHA (10∶1 to heme) and for the negative control DHA was omitted.

### Natural Product Library Screen

The 720 compound Natural Product Collection library (**Supporting Information S1**) from Microsource Discovery Systems, Inc. (Gaylordsville, CT, U.S.A.) was screened using the Heme Interaction Assay. Screening aliquots of compounds were obtained from the 10 mM stock plates by dilution to 1 mM using DMSO and dispensed into aliquot plates for storage at −80°C. A final concentration of 40 uM (2∶1 molar ratio to heme) was used for primary screening. To measure the power of the screening assay, Z-factor analysis [23] was performed (**Supporting Information S1**). Data from the primary screen were analyzed using Z score analysis [24] (**Supporting Information S1**). Compounds with a Z-score of ≥2, or ≤−2, were selected for follow-up analysis. The secondary screen consisted of repeating the heme interaction assay using an increased compound concentration of 200 uM (10∶1 molar ratio to heme) and the compounds with greater than a 10% A415 reduction were selected for further absorption spectral and cytotoxic analyses.

### Cell Culture and cytotoxicity assays

The lymphoblastoid leukemia cell line Molt-4 (ATCC CRL-1582), the breast cancer cell line MDA-MB-231 (ATCC HTB-26) and the prostate cancer cell line PC-3 (ATCC CRL-1435) were obtained from ATCC (Manassas, VA, USA). Molt-4 and MDA-MB-231 cells were maintained in RPMI-1640 medium, and PC-3 cells were cultured in F-12K medium, supplemented with 10% calf bovine serum in a humidified incubator with 5% CO_2_ at 37°C. Molt-4 cells were seeded at approximately 1×10^6^/ml followed by addition of compounds or equal volumes of DMSO and incubated for 24 hours or 48 hours. Compounds or equal volumes of DMSO were added to MDA-MB-231 and PC-3 cells at 60% confluence and incubated for 48 hours. DHA was used at 10 uM, 25 uM, or 40 uM, HTF at 12 uM, SA at 0.5 mM, ALA at 1 mM, and PPIX at 5 uM. Changes in cell number (proliferation) or cellular ATP level (cell viability) were analyzed. Live cells were distinguished from dead cells via Trypan Blue staining and counted using hemacytometer. The CellTiter-Glo Luminescent Cell Viability Assay [25] (Promega, WI, USA) was used to measure cellular ATP levels (Wallac 1420 multilabel counter).

### Statistical analysis

Results are presented as mean±s.d. for the indicated number of experiments. Statistical analyses were performed using one-way ANOVA and Student's t-test. A value of P<0.05 was considered to be statistically significant.

## Results

### Cytotoxicity of artemisinin depends on heme synthesis

**Figure 1:**
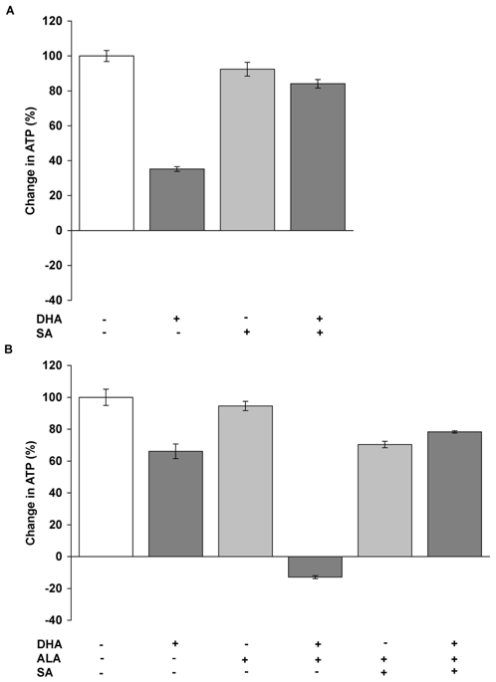
Modulation of heme synthesis alters DHA cytotoxicity in MOLT-4 cells. (A) Cellular viability of MOLT-4 cells cultured with DHA, succinyl acetone (SA), or both for 24 hours was measured using ATP levels. Values are mean +/− s.d. for six replicates. SA prevented DHA's cytotoxicity (p<0.001). (B) Cell viability of MOLT-4 cells cultured with DHA, aminolevulinic acid (ALA), and/or SA. ALA further increased the cytotoxicity of DHA (p<0.001), which was reversed by SA (p<0.001).

Previous studies have shown that leukemia cells are particularly sensitive to the artemisinin derivative dihydroartesunate (DHA) [26]. As an initial cell line to study, we selected MOLT-4 cells which have also been previously shown to be highly susceptible to DHA [27], [28]. Using this model for DHA cytotoxicity, we performed a dose response time course study (**Figure S1**) to identify a level of drug that resulted in significant cytotoxicity but not complete loss of the culture within 24 hours. A DHA concentration of 25 uM was selected. We then addressed the potential role of heme as the primary target for the cytotoxicity of artemisinin towards cancer cells by increasing or decreasing the rate of heme synthesis, which may be controlled pharmacologically. MOLT-4 cells were cultured with 25 uM DHA for 24 hours and cell viability measured via ATP content [25]. At 24 hours, the ATP level in the DMSO treated control cells increased by 119% (more than doubled) relative to time = 0, which was normalized to 100% in **Figure 1A**, and the cell number increased by 143% (normalized to 100% in **Figure S2A**). The ATP content in the culture treated with 25 uM DHA (**Figure 1A**) was only about 35% of the increase in ATP that was measured in control cells (p<0.05) while the increase in cell number (**Figure S2A**) was less than 30% of the increase of control cells (P<0.05). The smaller decrease in ATP content relative to the decrease in cell number measured via trypan blue exclusion is consistent with previous cytotoxicity studies [27]. To block heme synthesis and induce a relative heme deficiency, exogenous succinyl acetone (SA) was used to inhibit the enzyme aminolevulinate dehydratase to prevent the condensation of two molecules of ALA to form porphobilinogen and deplete the remainder of the heme synthetic pathway [29], [30]. The addition of SA by itself at 0.5 mM had no effect on cell viability or cell number, but essentially completely prevented cytotoxicity when co-incubated with DHA (**Figures 1A **and **S2A**). These results suggest that on-going heme synthesis may be essential for DHA cytotoxicity in Molt-4 cells. In addition, cellular heme levels of early log-growth cells do not appear to be sufficient to enable DHA cytotoxicity.

**Figure 2:**
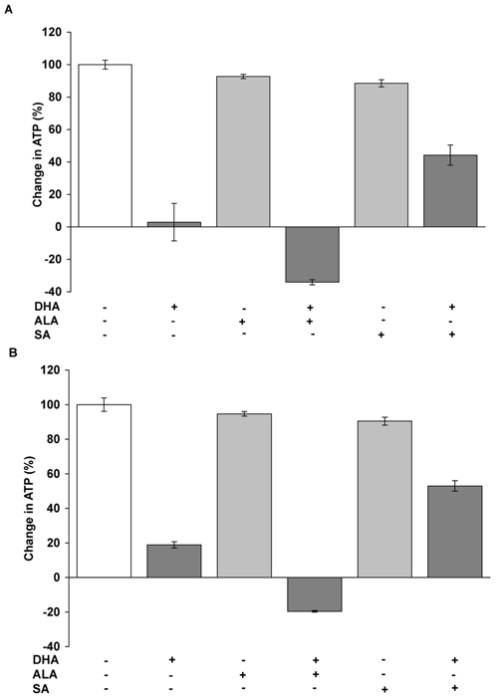
Modulation of heme synthesis alters DHA cytotoxicity in MDA-MB-231 and PC-3 cells. (A) Cell viability of MDA-MB-231 cells cultured with DHA, ALA, or SA. ALA further increased the cytotoxicity of DHA (p<0.001), which was largely reversed by SA (p<0.001). (B) Cell viability of PC-3 cells cultured with DHA, ALA, or SA. ALA also further increased the cytotoxicity of DHA (p<0.001), which was largely reversed by SA (p<0.001), similar to the effects seen with MDA-MB-231 cells.

We then tested whether increasing heme synthesis could potentiate the effects of DHA. To increase heme synthesis, exogenous aminolevulinic acid (ALA) was provided as a heme synthetic precursor, bypassing the ALA synthetase rate-limiting step and driving the synthetic pathway [31]. For this experiment, a lower concentration of DHA (10 uM) was used as a more stringent test for increased cytotoxicity. In this experiment, at 24 hours, the ATP level in the DMSO treated control cells increased by 111% relative to time = 0, which was normalized to 100% in **Figure 1B**, and the cell number increased by 112% (normalized to 100% in **Figure S2B**). The increase in ATP content of the culture exposed to 10 uM DHA for 24 hours was only 66% of the DMSO control, (**Figure 1B**) with a net loss of 4% in cell number from the initial culture (**Figure S2B**). Incubation of cells with 1.0 mM ALA by itself had no effect on the increase in ATP or cell number. However, co-incubation of 1.0 mM ALA with 10 uM DHA (**Figures 1B** and **S2B**) abrogated any increase in ATP content or cell number relative to control cells and resulted in a further decrease in ATP content (p<0.05), as well as a further loss in cell number relative to DHA treated cells (p<0.05). ALA appeared to significantly increase DHA cytotoxicity. To confirm this, SA was used to counter the effects of ALA because SA blocks an enzymatic step downstream from where ALA acts to increase heme synthesis. SA reversed the increased cytotoxicity of ALA and DHA and resulted in increases in ATP levels and cell numbers similar to controls (**Figures 1B** and **S2B**). These results suggest that ALA-induced heme synthesis can potentiate the cytotoxic effects of DHA, which could be blocked by the addition of SA to inhibit heme synthesis. In order to determine whether these effects were specific to Molt-4 cells, two cell lines of disparate tissue origin were analyzed, the breast cancer cell line MDA-MB-231 and the prostate cancer cell line PC-3. Both have been used in previous cytotoxicity studies involving artemisinin [32], [33]. Because these two cell lines are not as sensitive to the cytotoxic effects of artemisinin as are Molt-4 cells, a higher concentration of DHA (40 uM) was used and the incubation period was extended to 48 hours. DHA decreased cell viability, as expected, to a similar degree in both cell lines (**Figure 2**). Cell viability was further decreased in each of the cell lines by the addition of ALA, similar to the effect of ALA seen with Molt-4 cells. SA could then prevent most of the decrease in cell viability from DHA, although not to as great an extent as with Molt-4 cells. The mechanism for the lesser effect of SA in these cell lines is not clear, although the optimum dose of SA needed to maximally suppress heme synthesis has not been determined.

**Figure 3:**
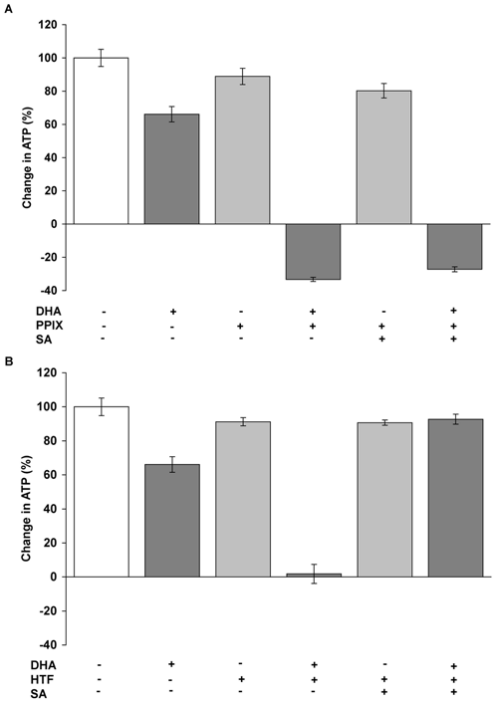
Effect of protoporphyrin IX and holotransferrin on DHA cytotoxicity. (A) Cell viability of MOLT-4 cells cultured with DHA, the heme precursor protoporphyrin IX (PPIX) and/or SA. PPIX increased DHA cytotoxicity (p<0.001), which could not be reversed by SA, consistent with PPIX acting later in the heme synthetic pathway than SA. (B) Cell viability of MOLT-4 cells cultured with DHA, holotransferrin (HTF), and/or SA. HTF enhanced DHA cytotoxicity (p<0.001), which was reversed by SA (p<0.001).

The modulation of heme synthesis with ALA does not exclude that other heme precursors may be mediating DHA cytotoxicity. To provide chemical specificity, protoporphyrin IX (PPIX), the penultimate molecule in the heme synthetic pathway into which a ferrous iron is inserted to generate heme, was used to increase heme synthesis[34]. PPIX at 5 uM had no significant effect on the increase in ATP content in the culture and caused a slightly decrease (p<0.05) in the cell number at approximately 84% of the increase in control cell number (**Figures 3A** and **S3A**). However PPIX dramatically increased the cytotoxicity from DHA, which resulted in 33% loss of ATP content (p<0.05) relative to control cells and a similar net loss of cell number (p<0.05) from the initial culture (**Figures 3A** and **S3A**). Co-incubation of PPIX and SA had no effect on the increase in ATP level or cell number, and when incubated with DHA, did not affect cytotoxicity (**Figures 3A** and **S3A**). This is consistent with PPIX acting further downstream in the heme synthetic pathway than SA. Because PPIX does not result in the activation of artemisinin in vitro [3], we attribute the effects of PPIX on the cytotoxicity of DHA to its stimulation of heme synthesis.

Another alternative explanation for the role of heme is that it is merely a means of increasing intracellular iron. To test this possibility, holotransferrin was added as an additional source of cellular iron [27]. As shown in **Figures 3B** and **S3B**, holotransferrin alone did not have a significant effect on the increases in ATP or cell number but dramatically increased the cytotoxic effect of DHA, an observation previously interpreted as due to the direct effects of iron [35], [36]. However, the newly acquired intracellular iron could also be used for heme synthesis. The addition of SA completely negated the effect of holotransferrin (**Figures 3B** and **S3B**), indicating that blocking heme synthesis can block the effect of iron supplementation via transferrin. These results suggest that non-heme iron contributes to the cytotoxicity of artemisinin through its use for heme synthesis.

### Spectrophotometric characterization of the Heme-Artemisinin interaction

**Figure 4:**
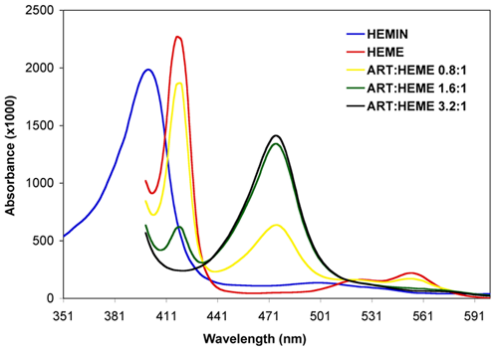
Absorbance at 415 nm is a sensitive indicator of the heme-artemisinin interaction. Absorption spectra of heme with increasing molar ratios of artemisinin to heme. A decrease in the Soret 415 nm absorbance peak occurs with the appearance of a peak at 476 nm.

With data supporting a primary role for heme in the mechanism of artemisinin's cytotoxicity towards human cancer cells, similar to its role in activating artemisinin in the killing of malaria parasites, we hypothesized that heme could serve as a potentially novel intracellular target for the development of both anti-neoplastic and anti-parasitic agents. In order to configure a high throughput assay, we used artemisinin (ART) as a model compound to characterize the interaction with heme using a simple absorption spectrum [37]. We have previously shown that artemisinin does not interact with hemin (oxidized heme) [3]. When hemin (Fe^3+^) was reduced to heme (Fe^2+^) by the addition of dithionite, a shift in the Soret peak absorbance [38] from 389 to 415 nm occurred (**Figure 4**). With the addition of artemisinin, the 415 nm Soret peak decreased and a peak at 476 nm appeared, as previously observed [37]. As the ratio of artemisinin to heme was increased, the 415 nm peak was incrementally decreased and was abrogated at very high drug∶heme ratios, while the peak at 476 nm increased further. The chemical species underlying the peak at 476 nm is not known but may be due to altered heme porphyrin ring spectral properties. Deformations of porphyrins are known to result in significant changes in the chemical and spectroscopic properties of the porphyrin ring. For example, properties of porphyrin rings can be modified through non-planar distortions such as altered oxidation potentials, modified basicity of the inner nitrogen atoms, and changes in axial ligand binding affinity [39]. Covalent modification of heme by artemisinin may also be a mechanism since artemisinin is known to alkylate heme [6].

### Sensitivity and specificity of the heme-artemisinin interaction

**Figure 5:**
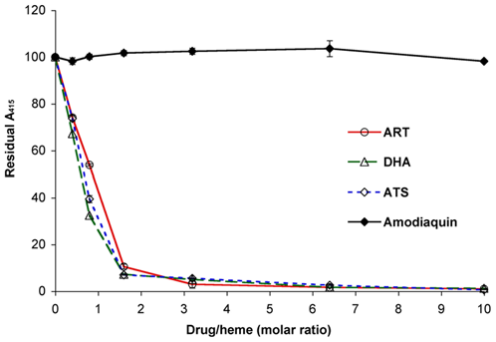
Sensitivity and specificity of detection of the heme-artemisinin interaction. Reduction of A415 peak absorbance was quantified with increasing drug∶heme ratios. Drug compounds include ART, DHA, and artesunate (ATS), and amodiaquin.

The sensitivity and specificity of detection were estimated using artemisinin, two related artemisinin derivatives, DHA and artesunate (ATS), as well as amodiaquine, a quinoline anti-malarial compound. Quinolines mediate their anti-malarial activity through interruption of hemin polymerization to prevent the formation of hemozoin that prevents toxicity from heme derived from ingested red blood cell hemoglobin. No effect on Soret band absorbance was observed from amodiaquine up to a ratio of 10∶1 with 20 uM heme (**Figure 5**). However, the assay was very sensitive for the detection of the artemisinin compounds, detecting a >20% decrease in A415 absorbance at an artemisinin drug∶heme ratio of 0.4∶1 with 20 uM heme (**Figure 5**).

We also addressed whether the endoperoxide moiety of artemisinin was a general mechanism for interaction with heme. A series of other compounds which possess peroxide groups including di-tert-amyl peroxide, tert-butyl peroxide, 2-butanone peroxide, dicumyl peroxide, benzoyl peroxide, lauroyl peroxide, and 9-α,11-α-di-hydroxy-5,8-epidioxy-5-α,8-α-ergostan-3-β-yl acetate (possesses an endoperoxide group) were not reactive in the assay at concentrations of 40 uM (**Figure S4**).

The potential effects of antioxidant compounds on the heme-artemisinin interaction were also tested. Butylated hydroxytoluene (BHT) and 6-hydroxy-2,5,7,8-tetramethylchroman-2-carboxylic acid (Trolox) up to 40 uM also had no effect (**Figure S5**).

### Heme Interaction High Throughput Screening (HI HTS) Assay

Hemin has been used as the target in previous high throughput screening studies. Approaches involving ultraviolet/visible spectrophotometry [40] and high performance liquid chromatography/diode array detector/mass spectrometry [41] have been proposed. While potentially useful as rapid, high throughput screening methods, such instrumentation may be costly, and are based upon physico-chemical interactions with hemin. We sought to develop a simple, robust microplate assay using absorption spectrometry based upon the interaction of compounds with heme (not hemin). However, conditions to prevent heme from spontaneously oxidized and allow for its solubility in aqueous solution while not interfering with the Soret absorption peak needed to be empirically determined. Our approach was to maintain heme in its reduced form using dithionite [42] and to use sufficient DMSO to maintain heme in its monomeric state [43], which also eliminated interference of absorbance from dithionite in overlapping wavelengths with the heme Soret band absorbance.

The decrease in Soret band absorbance at 415 nm that occurs when heme interacts with artemisinin was selected as a simple primary readout of a single endpoint assay, rather than using absorption spectra or focusing on the peak that occurs at 476 nm. The decrease in the Soret band absorbance will reflect a variety of alterations of the heme molecule and serves as a general indicator that a compound is interacting with heme. It is also a major change in the interaction with artemisinins. The peak observed at 476 nm may be unique to artemisinin. Other compounds may produce peaks at different wavelengths. Full wavelength spectra would therefore be useful to detect such new absorption peaks in secondary screens.

A simple and robust HTS 96 well microplate assay was developed to monitor heme's Soret band absorbance at 415 nm as a single endpoint assay. The assay includes the simple sequential steps of adding hemin, the chemical compound, and dithionite to reduce hemin to heme, followed by shaking and reading in a microplate reader. Parallel wells set up without heme are used to correct for A415 absorbance by compounds. The assay provides for a wide detection range, with an absorbance at 415 nm of about 2 absorbance units for 20 uM heme relative to a background empty well absorbance of about 0.10. Thus, the signal∶background ratio is approximately 20.

### Within-plate, plate-to-plate, and day-to-day assay variation

The variation in the performance of the assay was assessed using artemisinin. The coefficient of variation (CV) across an entire plate was 2–4%, whether measured as the variation of the assay without artemisinin (at about 2 absorbance units), or with a 8 uM∶20 uM artemisinin∶heme ratio (at about 1 absorbance unit).

The variation across microplates was similar to the within-plate variation at <5% (data not shown). The CV across different days was less than 8% (**Figure S6**). A Z-factor analysis [23] yielded a value of 0.701 on plate measurements performed on 5 consecutive days, indicating excellent performance. The main reagents used for the assay, hemin and DMSO, are stable chemicals and are very inexpensive, another advantage for high throughput screening. Only the dithionite stock solution was freshly prepared and all steps in the assay proceed sequentially with no incubations.

### Screening of a natural products library

**Figure 6:**
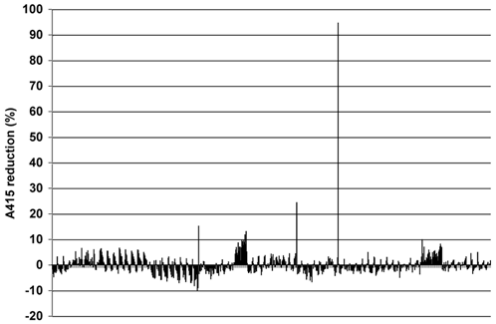
Primary screen of a natural products library for HI compounds. Percent reduction in A415 absorbance was plotted for each compound. The molar ratio of compound to heme was 2∶1 (40 uM∶20 uM).

The HI HTS assay was used to conduct an initial feasibility screen of a library of 720 compounds categorized as natural products [44]. Each compound was used in the microplate assay at a final concentration of 40 uM with heme at 20 uM. A primary screen yielded most compounds with little change in A415 (**Figure 6**).

Two criteria were used to select compounds for further analysis, z-score in the primary screen and an arbitrary threshold of >10% reduction in A415 in the secondary screen. For the primary screen, the compounds satisfying the criterion of z-score >2 or <−2 were subjected to a secondary screen at an increased compound concentration of 200 uM (10∶1 molar ratio to heme). In this secondary screen, only four compounds passed a threshold of >10% A415 reduction (**Figure S7**), including artemisinin (99% reduction in A415), coralyne (73% reduction in A415), 5α-cholestan-3β-ol-6-one (35% reduction in A415), and 3-hydroxy-4(succin-2-yl)-caryolane δ-lactone (11% reduction in A415). Artemisinin was one of the components of the commercial compound library. Consistent with the results for the effects of other antioxidants, nine antioxidant compounds contained in the natural products library including purpurogallin, silibinin, guaiazulene, guaiazulenel, chlorogenic acid, 4-acetoxyphenol, pomiferin, epicatechin, and pyrocatechuic acid, did not exhibit activity in the assay.

**Figure 7:**
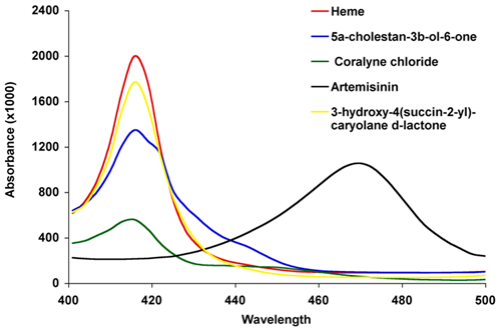
Absorption spectra of HI compounds. Absorption spectra of the four HI compounds were recorded over the wavelength range of 400 nm to 500 nm, which includes the heme Soret band absorption peak. The compound to heme molar ratio was 10∶1 (200 uM∶20 uM). Two of the four lead heme interacting compounds caused a substantial reduction in A415.

The reaction with heme of these four compounds at 200 uM was further analyzed using absorption spectra from 400 to 500 nm. Lower length wavelengths were not scanned because dithionite has a broad peak from ∼280 to 400 nm that would interfere. The 400 nm to 500 nm absorption spectrum of the interaction of heme and artemisinin displayed the characteristic disappearance of the 415 nm Soret band, as well as the previously described new peak at ∼476 nm (**Figure 7**). Coralyne also caused a dramatic decrease of heme Soret band absorbance but did not generate a new peak in the tested wavelength range. This is consistent with a significant structural change in the heme porphyrin ring structure, such as cleavage of the ring, and the generation of an intermediate or complex without absorbance characteristics. The compound 5α-cholestan-3β-ol-6-one caused a moderate reduction in heme Soret band absorption along with the generation of a new absorption peak(s) over-lapping the Soret 415 nm peak (right shoulder in **Figure 7**). The nature of the chemical species underlying this additional peak is not known. The compound 3-hydroxy-4(succin-2-yl)-caryolane δ-lactone was the least reactive compound and caused only a small reduction in heme Soret band without evidence of new absorption peaks.

### HI HTS compound cytotoxicity and dependence upon heme synthesis

**Figure 8:**
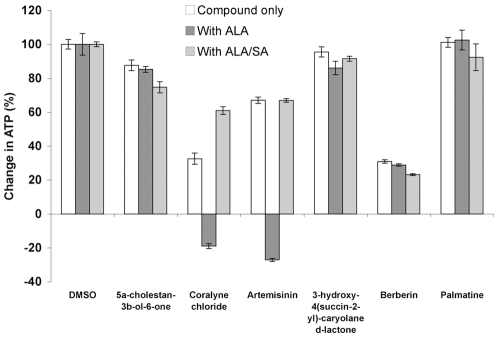
Heme synthesis modulates cytotoxicity of artemisinin and coralyne in MOLT-4 cells. The cytotoxicity of two heme interacting compounds can be modulated by increasing or decreasing heme synthesis. MOLT-4 cells were cultured for 48 hrs with the compounds shown, with or without ALA or ALA/SA.

The cytotoxic effects of the 4 lead compounds on the proliferation and viability of MOLT-4 cells was determined (**Figure 8**). Cells were seeded and exposed to compounds at 40 uM in the medium for up to 48 hours, a higher drug concentration than in previous experiments was used in order to detect cytotoxicity from weaker HI compounds. In this experiment, at 48 hours, the ATP content [25] in the DMSO control cells increased by over 200% relative to time = 0, which was normalized to 100% in **Figure 8**, with ATP level with ALA increasing by 140% and those exposed to ALA/SA increasing by 187%, both normalized to 100%. No impact on ATP levels was seen from 3-hydroxy-4(succin-2-yl)-caryolane δ-lactone. A small reduction in cell viability was seen with exposure to 5α-cholestan-3β-ol-6-one (an increase in ATP of 88%). Artemisinin had a modest impact on cell viability (an increase in ATP of 67%), in contrast to the strong effects seen with certain artemisinin derivatives, such as DHA used in **Figure 1**, consistent with previous reports of differential cytotoxicity among artemisinin compounds [45], [46]. DHA is the reduced lactol derivative of artemisinin, and semi-synthetic derivatives such as artemether, arteether, artesunate and artelinate are ethers or esters of the lactol. Differential solubility and/or chemical reactivity with heme may account for differences in cytotoxicity among these derivatives. The strongest cytotoxic effect of the 4 HI compounds was seen with coralyne (an increase in ATP of 33%), previously been shown to be an anti-cancer agent whose mechanism has been attributed to inhibition of topoisomerase I activity [47], [48], [49].

The effect of increasing heme synthesis on the cytotoxicity of HI HTS compounds was determined by co-incubation with 1 mM ALA. ALA had no effect upon of 5α-cholestan-3β-ol-6-one or 3-hydroxy-4(succin-2-yl)-caryolane δ-lactone. Co-incubation of ALA with artemisinin, however, caused a great reduction in cell viability, with a net 27% reduction in ATP content from the initial culture at time = 0. ALA also resulted in a dramatic reduction in cell viability when co-incubated with coralyne, with a net loss in ATP content of 19%. These results suggest that the mechanism of cytotoxicity due to coralyne may be in part dependent upon heme.

Inhibition of heme synthesis by SA was used to reverse the effects of ALA. Incubation with SA/ALA had little effect upon cell viability with 3-hydroxy-4(succin-2-yl)-caryolane δ-lactone and a slight decrease in cell viability with 5α-cholestan-3β-ol-6-one relative to incubation with ALA without SA. We conclude that modulation of heme synthesis has little effects upon cytotoxicity from these two compounds, consistent with their much lower reactivity towards heme than either artemisinin or coralyne.

In contrast, the presence of SA completely prevented the increase in artemisinin's toxicity from ALA and resulted in cell viability similar to artemisinin alone. SA also prevented the increased cytotoxic effects of coralyne by ALA and resulted in a cell viability level even higher than the culture treated only with coralyne. We conclude that modulation of heme synthesis has significant effects upon cytotoxicity from artemisinin and coralyne, consistent with their high reactivity towards heme in vitro. These data further support heme as a molecular target for cytotoxicity.

**Figure 9:**
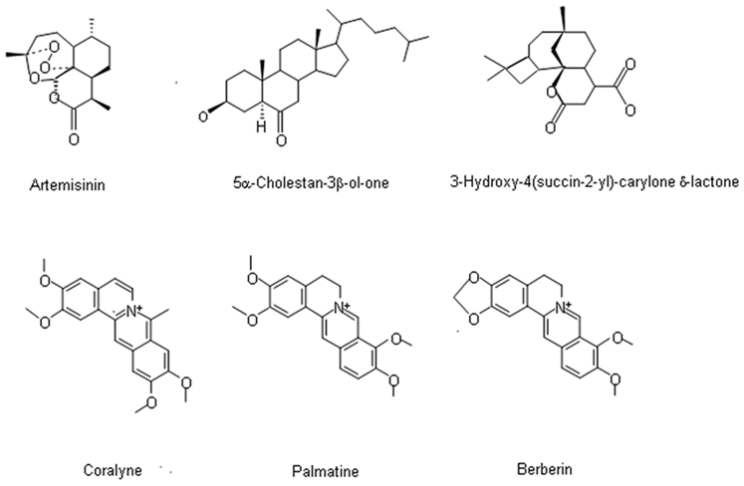
Structures of lead heme interacting compounds and structurally related compounds.

The HI HTS assay appears to distinguish among slight differences in structure. The compound palmatine, also present in the compound library and which shares high structural similarity with coralyne (**Figure 9**), does not react with heme and shows no toxicity towards Molt-4 cells (**Figure 8**). Berberin, another close structural relative of coralyne present in the compound library, also possesses cytotoxic activity [50], [51] but did not react in the HI HTS assay. Berberin at 40 uM exhibited strong cytotoxicity against Molt-4 cells (**Figure 9**). In contrast to coralyne, ALA appeared to be somewhat protective, while SA slightly increased cytotoxicity (**Figure 9**). A major structural difference that distinguishes coralyne from both palmatine and berberin is the presence of a fully aromatic ring structure that is disrupted in palmatine and berberin due to reduction in the nitrogen-containing ring. Therefore a planar configuration and broad dislocation of π-electrons in coralyne are not present in palmatine and berberin, which may account for their inaccessibility and/or chemical non-reactivity with heme. The interaction of coralyne with heme may be due to the favorable planar ring structure which may allow for physical association and a strong stacking interaction.

## Discussion

Artemisinins exhibit both anti-malarial activity and selective cytotoxicity against cancer cells. We previously have shown that heme is the kinetically favored iron species that can react with artemisinin [3]. We show here that heme is also involved in the mechanism of the cytotoxic effects of artemisinins in at least three different cell lines, suggesting that this may be a generalizable mechanism. Heme is an essential cellular molecule whose redox properties and participation in a variety of important cellular functions render it a potentially valuable molecular target for the development of anti-neoplastic agents. The essential role of heme in mediating the anti-malarial activity of artemisinin drugs, and the dual effectiveness of the artemisinin compounds against malaria parasites and cancer cells, is consistent with our findings that heme plays a similar role in mediating cytotoxicity [9]. Cancer cells have been shown to be much more sensitive to artemisinins than their normal counterparts [27], [36], and cancer cell lines with a low percentage of G0/G1 cells and a high percentage of S-phase cells have been found to be most sensitive to artemisinin drugs, consistent with the relatively increased rate of heme synthesis needed for cell division [9]. Cancer cells also differ significantly from normal cells in their heme synthetic capacity [19], which is the basis for several types of photodynamic therapies for cancer [21], [22].

We used a simple assay to identify other heme interacting compounds. The mechanism by which these compounds interact with heme may vary. Heme is comprised of a coordination complex formed between iron and the dianionic form of porphyrin, a heterocyclic macrocycle derived from 4 pyrrole subunits linked on opposite sides through 4 methine bridges. The porphyrin ring is very hydrophobic and may promote interactions with other hydrophobic molecules. Heme's side chains may also provide a means for interaction. Several forms of heme exist depending upon the modification of side chains. The free form, heme b, contains 4 methyl groups, two propionic side chains, and two vinyl groups which can be modified for covalent linkage to proteins.

While these hydrophobic and/or side chain interactions may contribute, iron moiety of heme plays a critical role. Heme iron is coordinated to 4 pyrrole nitrogens within the pyrrole ring. The fifth and sixth coordination positions can be liganded to proteins or other molecules. Due to the coordinated iron, heme is a strong reducing agent which may contribute to the interaction with HI compounds, which could produce chemical disruption of heme's ring structure and loss of Soret band absorbance. The large reduction of the Soret band A415 from coralyne, without the appearance of any other spectroscopically detectable species, is consistent with breakage of the heme ring structure, analogous to the cleavage of DNA by coralyne [52]. The interaction of artemisinins with heme appears to procede through several intermediates as evidenced by new but unstable absorption peaks. The initial new peak that occurs at 476 nm may be an intermediate complex between heme and artemisinin radicals [37], which is not stable and disappears [3], consistent with decomposition of heme porphyrin ring.

Little is known about the molecular mechanisms responsible for HI compound cytotoxicity. The mechanisms by which artemisinins are selectively cytotoxic to cancer cells have been suggested to include increasing reactive oxygen species and inhibiting hypoxia inducible factor (HIF) alpha activation [53]. HIF is the primary transcription factor that regulates gene expression in response to hypoxia. During hypoxia, HIF-alpha levels accumulate and trigger an increase in expression of genes involved in energy metabolism and mitochondrial function, cell survival and apoptosis, and resistance to oxidative stress [54], physiological processes that require heme. HIF may also be involved in the inhibition of tumor lymphangiogenesis by artemisinin through suppression of vascular endothelial growth factor C [16]. Artemisinin has also been reported to activate p38 mitogen-activated protein kinase (p38 MAPK) in human lung cancer cells [18]. HO-1, the primary enzyme that metabolizes heme, can be significantly inhibited by p38 MAPK inhibitors [55].

Coralyne and several of its structural analogues have been shown to be inhibitors of DNA topoisomerase I, with structural rigidity associated with the coralyne ring system thought to be important for its pharmacological activity [47], [48]. Palmatine and berberin, which share significant structural similarity with coralyne, are also DNA-binding alkaloids [50], [51], although they are not reactive with heme nor is their cytotoxicity affected by heme synthesis as is coralyne. The binding of coralyne and palmatine with nucleic acid in vitro may involve a substantial hydrophobic interaction [50]. The identification of coralyne but not its structural analogs as heme interacting compounds suggests that its mechanism of cytotoxicity may be more complex.

The two most potent HI compounds, artemisinin and coralyne, also both possess activity against parasites [56], [57], [58] and cancer cells [49], [59]. The dependence on heme synthetic activity for cytotoxicity suggests a general mechanism for the pharmacological function of these HI compounds in treating protozoan infections. Analogous to cancer cells that exhibit high rates of proliferation, malaria parasites at the intraerythrocytic stage replicate rapidly and are highly susceptible to artemisinins through a mechansism thought to involve heme derived from red blood cells [60]. However, malaria parasites produce heme by de nove synthesis [61], [62]. Our results suggest that artemisinins may interact with parasite derived heme, which would explain why artemisinins can effectively kill malaria parasites in host erythrocytes where the hemoglobins are poisoned by carbon monoxide and the heme iron is not available [63]. A similar mechanism may also exist for other protozoan parasites, such as Leishmania, which are able to synthesize heme from iron and protoporphyrin [64]. This is also supported by the observation that the ferrous iron transporter of Leishmania (LIT) is essential for parasite replication within macrophages [65], [66], suggesting that Leishmania parasites rely on endogenous rather than exogenous heme that could interact with the Leishmaniacides artemisinins [67], [68], [69] or coralyne [56], [57].

## Supporting Information

Figure S1 Time course and dose response of DHA on Molt-4 cells. The proliferation of Molt-4 cells was significantly decreased by concentrations of DHA exceeding 25 uM.
(0.12 MB TIF)

Figure S2 Modulation of heme synthesis alters DHA cytotoxicity in Molt-4 cells. Cell numbers were determined at the beginning and the end of treatment, and the change during the treatment was expressed as percentage, taking DMSO control as 100%. The data are presented as Mean±SD. (A) Cellular proliferation of Molt-4 cells cultured with DHA, succinyl acetone (SA), or both, for 24 hours was measured using cell number. Values are mean +/− s.d. for six replicates. SA resulted in a statistically significant (p<0.05) increase in cell number. (B) Cell numbers of Molt-4 cells cultured with DHA, aminolevulinic acid (ALA), and/or SA. ALA further increased the cytotoxicity of DHA (*p<0.05), which was reversed by SA.
(1.50 MB TIF)

Figure S3 Effect of protoporphyrin IX and holotransferrin on DHA cytotoxicity. (A) Cell numbers of Molt-4 cells cultured with DHA, holotransferrin (HTF), and/or SA. HTF enhanced DHA cytotoxicity (*p<0.05), which was reversed by SA. (B) Cell proliferation of Molt-4 cells cultured with DHA, the heme precursor protoporphyrin IX (PPIX) and/or SA. PPIX increased DHA cytotoxicity (*p<0.05), which could not be reversed by SA, consistent with PPIX acting later in the heme synthetic pathway than SA.
(1.50 MB TIF)

Figure S4 Reduction in absorbance at 415 nm in the presence of various peroxides. Each peroxide compound was at 40 uM with heme at 20 uM in dithionite sodium phosphate buffer solution with 36% DMSO. Di-T-A =  di-tert-amyl peroxide; tert-B =  tert-butyl peroxide; 2-But =  2-Butanone peroxide; Di-Cum =  Dicumyl peroxide; Benz =  Benzoyl peroxide; Laur =  Lauroyl peroxide; 5,8-Epi =  9-α,11-α-di-hydroxy-5,8-epidioxy-5-α,8-α-ergostan-3-β-yl acetate; DHA =  Artesunate.
(0.10 MB TIF)

Figure S5 Reaction between ferrous heme and artemisinins in the presence of trolox (TLX) or BHT. Absorption spectra were recorded over the wavelength range of 400 nm to 500 nm, which shows that neither TLX nor BHT had any effect on the interaction of heme and ART.
(0.32 MB TIF)

Figure S6 Day-to-day variation of the Heme Interaction assay. Assays were conducted using an entire microplate for five consecutive days. The ratio of artemisinin to hemin was 0.8∶1.
(0.13 MB TIF)

Figure S7 Secondary screen of the compounds selected from primary screen. Compounds were used at 200 uM (10∶1 molar ratio to heme). Four compounds caused a 10% or more reduction in A415 including artemisinin, coralyne, 5α-cholestan-3β-ol-6-one and 3-hydroxy-4(succin-2-yl)-caryolane δ-lactone.
(0.09 MB TIF)

Supporting Information S1 Methods
(0.03 MB DOC)
