# Supplementary figures and images for: Heme Mediates Cytotoxicity from Artemisinin and Serves as a General Anti-Proliferation Target
Source: PLoS One. 2009 Oct 28;4(10):e7472. doi: 10.1371/journal.pone.0007472 (PMC2764339; doi:10.1371/journal.pone.0007472)

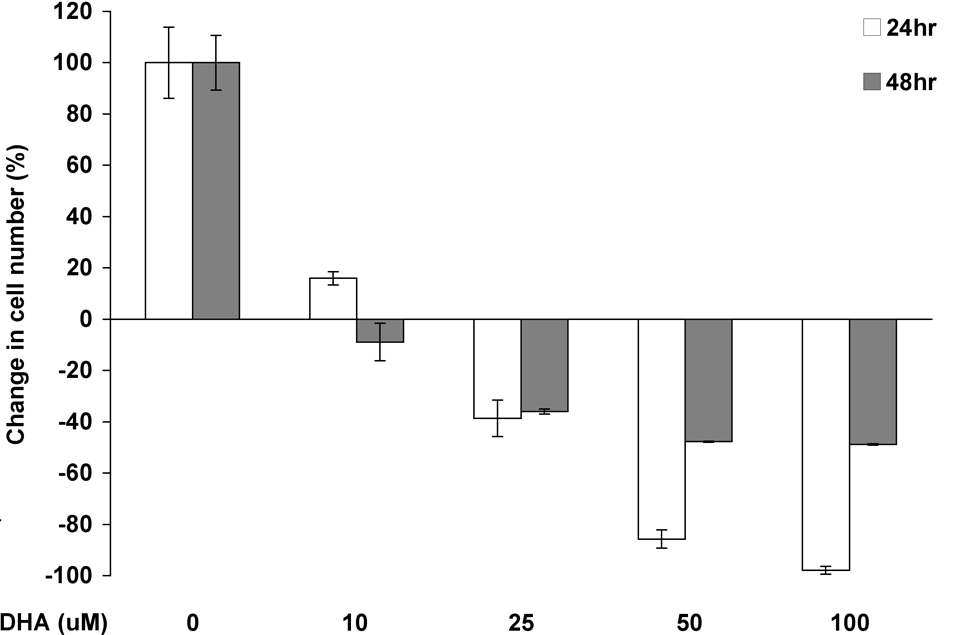

Supplement: Figure S1 — Time course and dose response of DHA on Molt-4 cells. The proliferation of Molt-4 cells was significantly decreased by concentrations of DHA exceeding 25 uM. (0.12 MB TIF) [file pone.0007472.s001.tif]

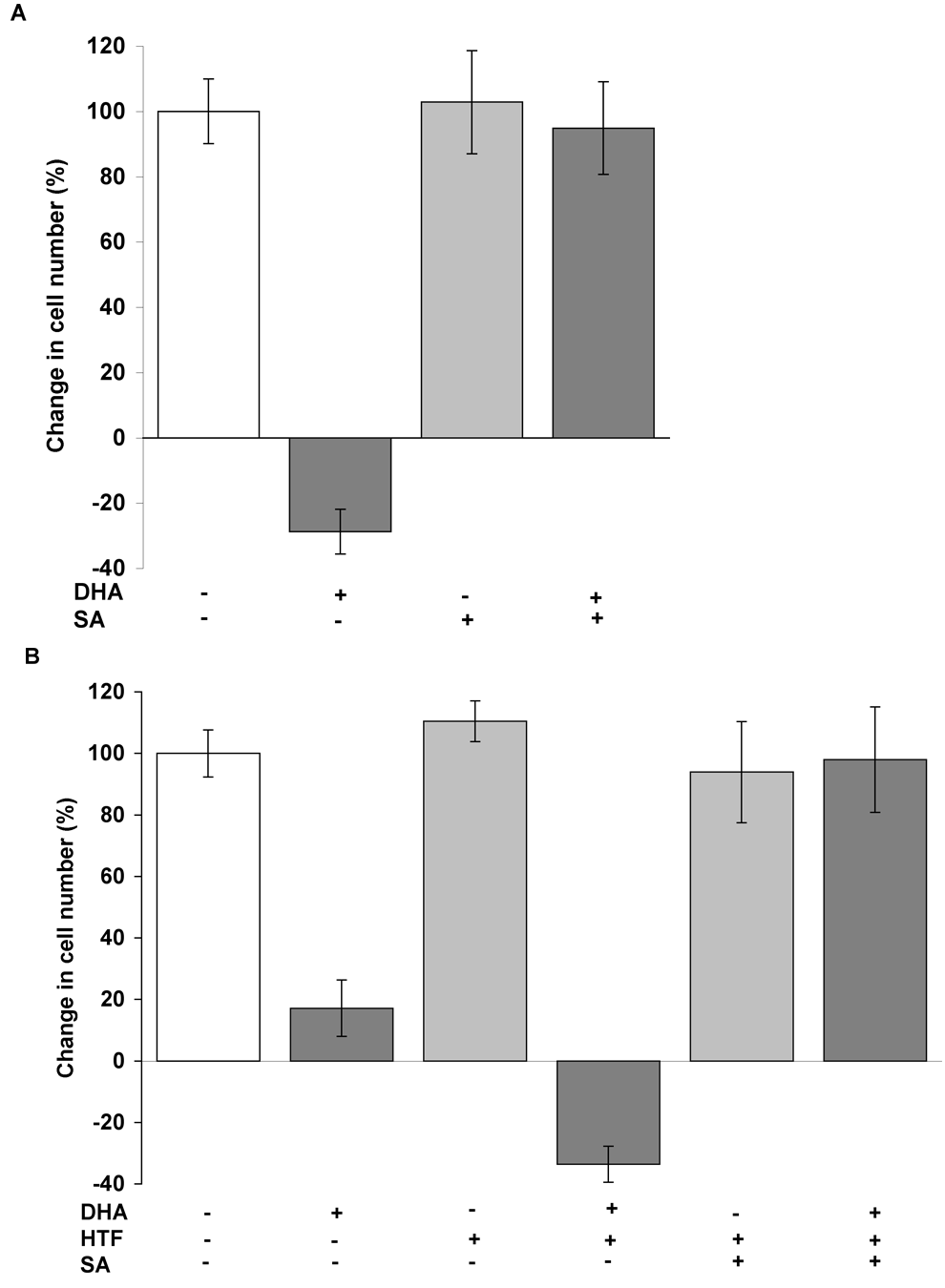

Supplement: Figure S2 — Modulation of heme synthesis alters DHA cytotoxicity in Molt-4 cells. Cell numbers were determined at the beginning and the end of treatment, and the change during the treatment was expressed as percentage, taking DMSO control as 100%. The data are presented as Mean±SD. (A) Cellular proliferation of Molt-4 cells cultured with DHA, succinyl acetone (SA), or both, for 24 hours was measured using cell number. Values are mean +/− s.d. for six replicates. SA resulted in a statistically significant (p<0.05) increase in cell number. (B) Cell numbers of Molt-4 cells cultured with DHA, aminolevulinic acid (ALA), and/or SA. ALA further increased the cytotoxicity of DHA (*p<0.05), which was reversed by SA. (1.50 MB TIF) [file pone.0007472.s002.tif]

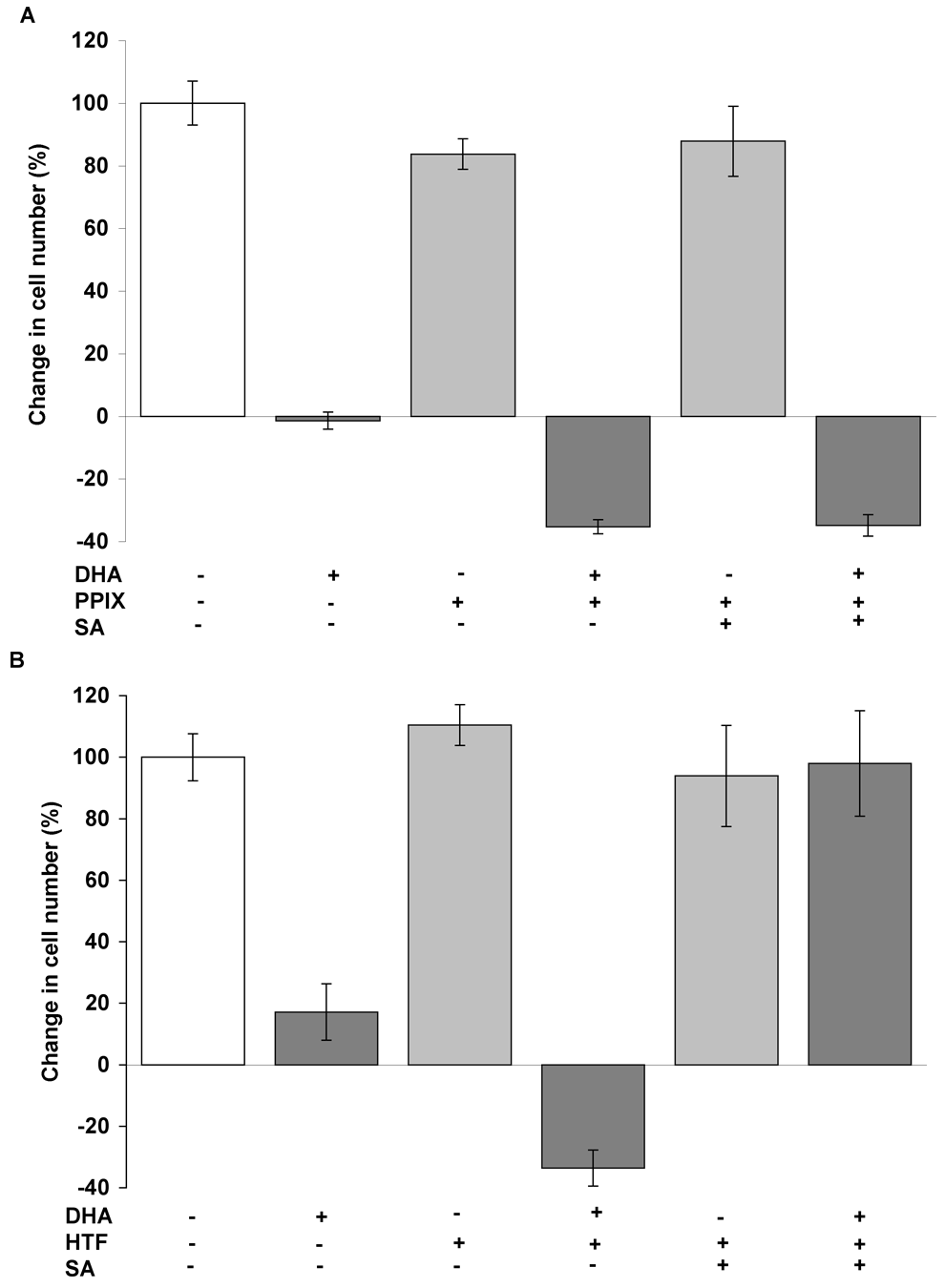

Supplement: Figure S3 — Effect of protoporphyrin IX and holotransferrin on DHA cytotoxicity. (A) Cell numbers of Molt-4 cells cultured with DHA, holotransferrin (HTF), and/or SA. HTF enhanced DHA cytotoxicity (*p<0.05), which was reversed by SA. (B) Cell proliferation of Molt-4 cells cultured with DHA, the heme precursor protoporphyrin IX (PPIX) and/or SA. PPIX increased DHA cytotoxicity (*p<0.05), which could not be reversed by SA, consistent with PPIX acting later in the heme synthetic pathway than SA. (1.50 MB TIF) [file pone.0007472.s003.tif]

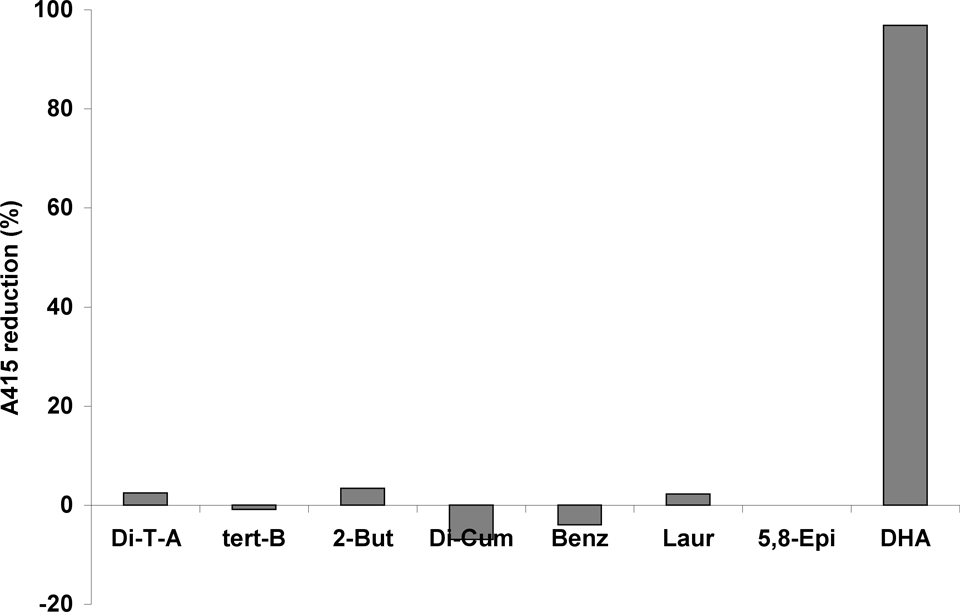

Supplement: Figure S4 — Reduction in absorbance at 415 nm in the presence of various peroxides. Each peroxide compound was at 40 uM with heme at 20 uM in dithionite sodium phosphate buffer solution with 36% DMSO. Di-T-A = di-tert-amyl peroxide; tert-B = tert-butyl peroxide; 2-But = 2-Butanone peroxide; Di-Cum = Dicumyl peroxide; Benz = Benzoyl peroxide; Laur = Lauroyl peroxide; 5,8-Epi = 9-α,11-α-di-hydroxy-5,8-epidioxy-5-α,8-α-ergostan-3-β-yl acetate; DHA = Artesunate. (0.10 MB TIF) [file pone.0007472.s004.tif]

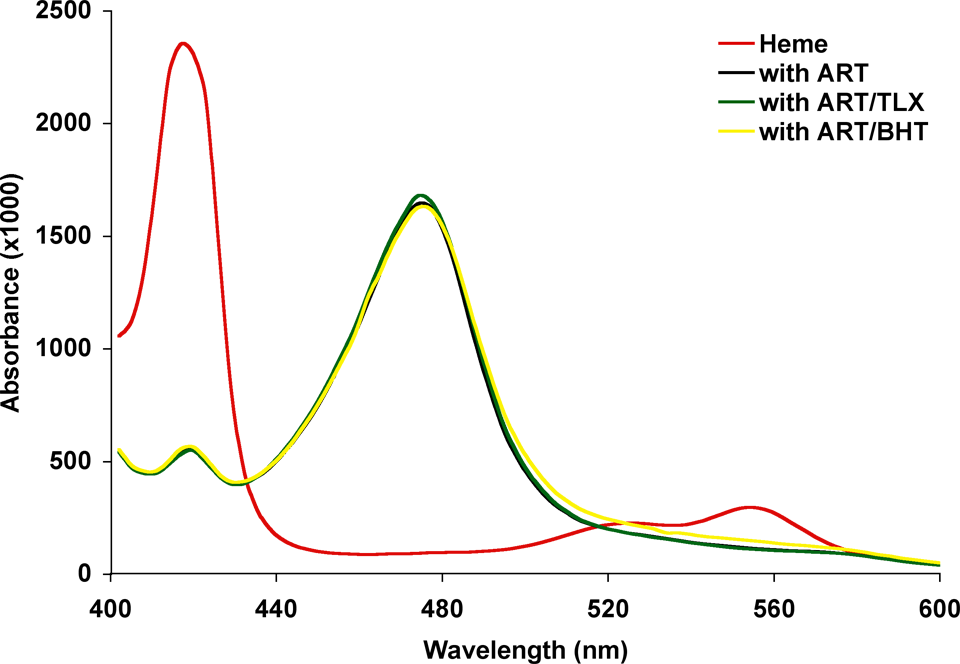

Supplement: Figure S5 — Reaction between ferrous heme and artemisinins in the presence of trolox (TLX) or BHT. Absorption spectra were recorded over the wavelength range of 400 nm to 500 nm, which shows that neither TLX nor BHT had any effect on the interaction of heme and ART. (0.32 MB TIF) [file pone.0007472.s005.tif]

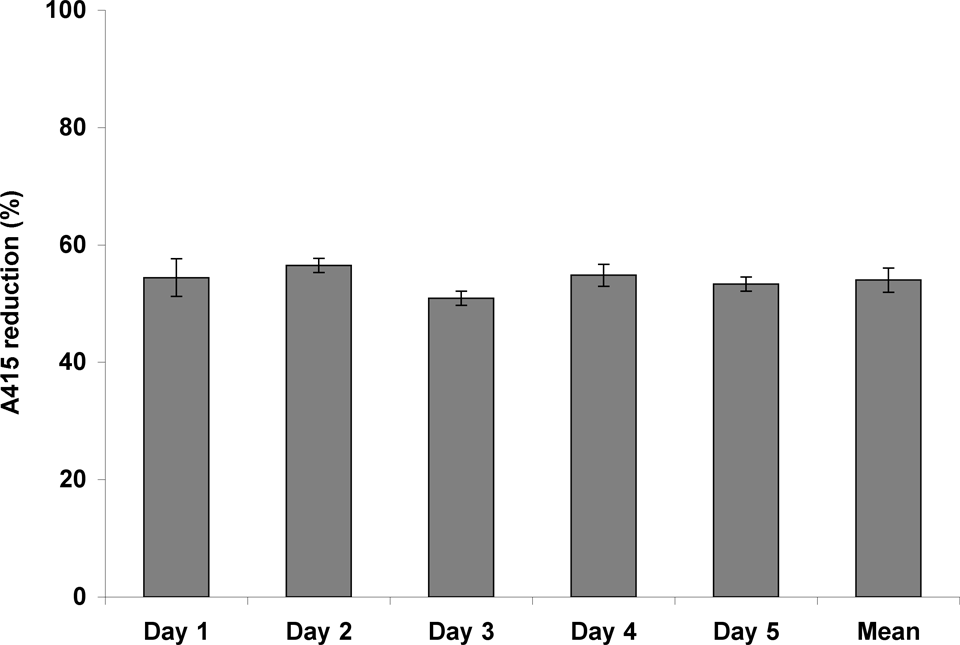

Supplement: Figure S6 — Day-to-day variation of the Heme Interaction assay. Assays were conducted using an entire microplate for five consecutive days. The ratio of artemisinin to hemin was 0.8∶1. (0.13 MB TIF) [file pone.0007472.s006.tif]

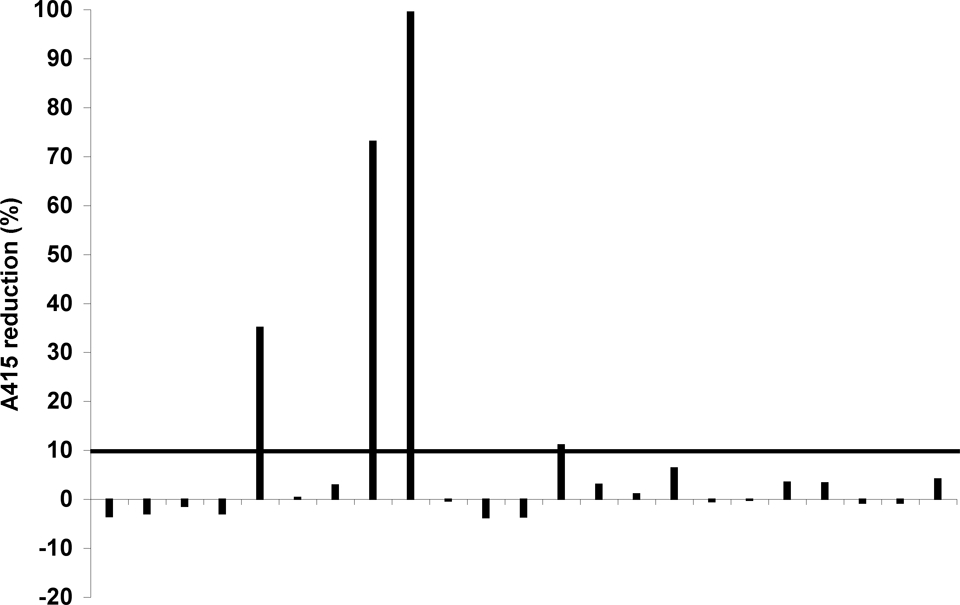

Supplement: Figure S7 — Secondary screen of the compounds selected from primary screen. Compounds were used at 200 uM (10∶1 molar ratio to heme). Four compounds caused a 10% or more reduction in A415 including artemisinin, coralyne, 5α-cholestan-3β-ol-6-one and 3-hydroxy-4(succin-2-yl)-caryolane δ-lactone. (0.09 MB TIF) [file pone.0007472.s007.tif]
