# Supplementary material for: Heme Mediates Cytotoxicity from Artemisinin and Serves as a General Anti-Proliferation Target
Source: PLoS One. 2009 Oct 28;4(10):e7472. doi: 10.1371/journal.pone.0007472 (PMC2764339; doi:10.1371/journal.pone.0007472)
Supplement: Supporting Information S1 — Methods (0.03 MB DOC) [file pone.0007472.s008.doc]

**Methods**

**Absorption Spectra**

Absorbance spectra were recorded from total volume of 1ml in a plastic cuvette using a Spectronic Genesys 5 (Spectronic Instruments, Inc Rochester, NY). The molar ratio of compound:heme was 2:1.

**Natural Product Library Screen.**

This natural product collection includes compounds derived from materials obtained from botanical exporters worldwide including Chile, China, India, Peru, Russia, and North America. All compounds were at a minimum 95% purity and were selected on the basis of structural and chemical class. Approximately 75% of the collection includes alkaloids (16%), flavonoids (12%), sterols/triterpenes (12%), diterpenes/sesquiterpenes (10%), benzophenones/chalcones/stilbenes (10%), limonoids/quassinoids (9%), and chromones/coumarins (6%). The remainder of the compounds include quinones/quinonemethides, benzofurans/benzopyrans, rotenoids/xanthones, carbohydrates, and benztropolones/depsides/depsidones.

Z-factor analysis, a measure of the quality or power of a high-throughput screening assaywas calculated as one minus the following: three times the sum of the positive and negative control standard deviations divided by the absolute value of the difference between the positive and control means. The Z-factor was interpreted as ideal (1.0), excellent (0.5-1.0), marginal (0-0.5), and unusable (<0).

The Z score was calculated by subtracting the average of all compound measurements on a plate from each individual compound measurement and divided by the standard deviation of all compound measurements on the plate.
